# Polio Survivors’ Experiences of Corrective Surgery and Rehabilitation in 1950s–1960s Finland 

**DOI:** 10.1177/23333936261480474

**Published:** 2026-08-21

**Authors:** Minna Elomaa-Krapu

**Affiliations:** 1 Faculty of Social Sciences, Nursing Science, 7840 Tampere University, Tampere, Finland; 2 RDI & Education, Wellbeing Services County of Vantaa and Kerava, Vantaa, Finland

**Keywords:** poliomyelitis, rehabilitation, surgery, child, qualitative research, Finland

## Abstract

This study aimed to explore polio survivors’ childhood experiences of corrective surgery and rehabilitation in post-war Finland during the 1950s and 1960s. Oral history research was used with a descriptive qualitative approach. Between September 29, 2018 and June 30, 2019, 40 participants were interviewed, while one wrote about their experiences (N = 41). The data were analyzed using reflexive thematic analysis, which produced the overarching theme ‘The Ongoing Process of Recovery: Body, Mind, and Self, ‘describing recovery as a holistic process shaped by bodily changes, emotional responses, and social experiences. Three related themes further elaborated children’s treatment trajectories: ‘Surgical Treatments as Healing and Traumatizing Experiences,’ ‘The Multidimensional Nature of Rehabilitation,’ and ‘The Child’s Emotional Coping and Agency in the Hospital Environment.’ The findings emphasize that corrective surgery and rehabilitation were not experienced solely as clinical interventions but as life-shaping events influencing children’s sense of safety, control, and self. This study highlights the need to consider the long-term psychosocial impact of childhood hospitalization and supports child-centered, family-centered, and trauma-informed approaches in pediatric care.

## Introduction

Poliovirus is an RNA virus transmitted via contact or droplet infection. It enters through the alimentary canal and into the bloodstream. While some infected individuals remain asymptomatic, its common symptoms include fever, headache, fatigue, stiffness in the neck or back, and pain in the limbs. No cure exists for polio, but its symptoms can be treated, and vaccination is the most effective means of its prevention (Khan, 2010; Oshinsky, 2005; Trevelyan et al., 2005). Polio persists in various Asian and African countries, and the World Health Organization continues to recommend vaccination (World Health Organization, 2021). Polio transmission surged across North America and Europe in the early 1940s, with its incidence peaking in the 1940s and 1950s (Alaranta et al., 2002; Altenbaugh, 2015; Nathanson & Kew, 2010; Rutty, 1996; Trevelyan et al., 2005). In Finland, between 1954 and 1956, approximately 700 to 800 cases were detected annually, and the total number of infected individuals was estimated at 10,000 (Valtonen et al., 2005). In 1957, vaccination campaigns began, eradicating the disease in the 1960s, but it re-emerged in 1984, with the most recent case reported in 1985 (Finnish Institute for Health and Welfare, 2019; Muiluvuori, 2010).

The onset of polio infection was sudden and its progression unusual. Since its symptoms—stiff neck, fever, nausea, and lethargy—overlapped with those of other pediatric illnesses, diagnosis was challenging. Some infected individuals became paralyzed, as the nerve cells in their spinal column were damaged and destroyed, while others died from respiratory failure. The difficulty of diagnosis and uncertainty about the origin and transmission of polio generated widespread fear (Carter, 2001; Highley, 2016; Wilson, 2005). The children and adolescents diagnosed with polio had to be immediately isolated—in hospitals, infectious disease units, or at home—throughout the acute phase of the disease, which was when the contagion risk was highest. The isolation stage lasted approximately two weeks, but the acute phase lasted totally approximately four weeks. Immediately afterward, the patients entered the challenging rehabilitation phase in the hospital or at home, which, depending on the damage wreaked on their bodies, lasted for weeks or years. Some suffered respiratory paralysis and were placed on ventilators, commonly known as iron lungs (Altenbaugh, 2015; Muiluvuori, 2010; Wilson, 2005).

Following the acute and isolation phases, the patients with polio underwent prolonged orthopedic and rehabilitation treatments. To support function and correct deformities, plaster applications, casts, physiotherapy, and orthotic devices were used. When conservative treatment proved insufficient, corrective surgical procedures—including tendon transfers, joint fusions, and epiphysiodesis to halt asymmetric bone growth—were performed. Since children’s bodies and physical autonomy were placed entirely in the hands of others, it compounded the experiences of trauma, loss of identity and control, and social isolation (Altenbaugh, 2015; Highley, 2016).

 The experience of bodily objectification and loss of agency has been theorized through Foucauldian frameworks as a process of designation and division, in which a child’s disabled polio body is subjected to medical and rehabilitative discourses that effectively silence the child’s lived experience (Yoshida & Shanouda, 2015). Polio also profoundly disrupted children’s access to education and schooling, with many children relegated to special classes, denied re-entry to school, or offered only home-based instruction. Moreover, prolonged treatment increasingly blurred the boundaries between hospital, home, and school (Altenbaugh, 2006).

Rehabilitation methods changed considerably worldwide during the epidemic period. Early treatment relied on immobilization using splints and plaster casts, which often atrophied muscles and worsened deformities. A fundamental shift occurred internationally in the early 1940s, when immobilization as the dominant treatment philosophy was gradually replaced by the Kenny method, which involved hot packs, gentle muscle exercises, and active patient participation in recovery (Neumann, 2004; Rogers, 2021).

Finland did not widely adopt the Kenny method. Considered extremely taxing for the patient, the method was not recommended by the nursing textbooks of the 1950s and 1960s, and polio treatment continued to follow the prevailing nurse-centered, paternalistic care focused on symptom management (Veltheim et al., 2024). Although patient-centered care was beginning to develop, professional attitudes remained distant, and nurses’ limited awareness of children’s developmental, social, and psychological needs biased care heavily toward physical and medical concerns (Veltheim et al., 2024; Wilson, 2005). Parental presence was not considered favorably in pediatric hospital settings, and family-centered care developed slowly (Jolley & Shields, 2009). Strict hospital routines and visitor restrictions augmented children’s loneliness, and limited contact with home and family was justified by the belief that children would otherwise become disobedient (Highley, 2016). As previous Finnish research has shown, polio survivors recall the isolation phase as lonely and silent, with care perceived as cold and dismissive and children excluded from decisions about their own treatment (Elomaa-Krapu & Kaunonen, 2023). According to international research on polio survivors, children were deeply unsettled and saddened when they were sent away from home for specialized treatment; sometimes their parents and relatives could not visit them at all due to the distance between home and clinic (Sjödahl Hammarlund et al., 2021). The physical, psychological, and social suffering of young polio survivors has not been fully acknowledged, and their childhood experiences of corrective surgery and rehabilitation have not been sufficiently examined globally from a nursing perspective (Altenbaugh, 2015; Highley, 2016).

The historical origins of family-centered care are rooted in the suffering that followed World War II; in the mid-twentieth century, parents worldwide were often prevented from visiting their hospitalized children altogether or were only permitted half-an-hour-per-week visits (Jolley & Shields, 2009). The philosophy of pediatric nursing has since advanced considerably and is now grounded in family-centered care, which emphasizes the dynamic relationship between child patients and their families, the importance of maintaining normal family life, and the reduction of children’s stress (Kuo et al., 2012; Shields et al., 2012).

The present research forms part of a broader research program examining the experiences of child polio survivors and their siblings across the illness and treatment trajectory and the wider impact of the disease on families. The research program—the first nursing study to document polio survivors’ experiences in Finland during the post-war decades of the 1950s and 1960s—aims to contribute to the historical literature on pediatric care, the sequelae of polio and paralysis, and the social significance of these experiences in Finnish society. A secondary aim is to enrich understanding of the history of care provided to polio-affected children and families. Two earlier articles from this research program have been published: The first addressed children’s experiences during the isolation phase (Elomaa-Krapu & Kaunonen, 2023), and the second reported experiences from the post-isolation acute phase prior to discharge or transfer to a rehabilitation facility (Elomaa-Krapu & Kaunonen, 2024). The present article focuses on corrective surgery and rehabilitation.

## Method

### Aim

This study aims to understand polio survivors’ childhood experiences of corrective surgery and rehabilitation in post-war Finland during the 1950s and 1960s. These experiences were situated in hospitals, children’s hospitals, and specialist rehabilitation centers. The treatment encompassed diverse rehabilitative interventions, including practicing the movement of paralyzed limbs, relearning to walk, and other physical exercises aimed at restoring functional capacity, as well as corrective surgical procedures, such as tendon transfers, joint fusions, and epiphysiodesis performed to halt asymmetric bone growth. This article does not address self-directed rehabilitation conducted at home.

### A Descriptive Oral History

Oral history research was conducted using a descriptive qualitative approach (Doyle et al., 2020), which guided data collection and the systematic progression of the analysis, while an interpretative oral history framework and a constructivist approach (Abrams, 2010) directed the interpretation of the data and the understanding of the constructivist nature of memory. The two approaches thus complemented one another across a process spanning from data collection to interpretation. As Nelson (2002) argued, nursing history should not be treated merely as one qualitative method among others, but pursued rigorously as a discipline requiring both nursing and historical expertise.

Oral history is an interactive process in which meaning is co-constructed between the researcher and narrator through dialogue (Abrams, 2010). Its key strength is giving voice to groups outside conventional historiography, with the aim of accessing individual experiences rather than producing a definitive history (Shopes, 2011; Thompson, 2000). Recollections are shaped by the intersubjective dynamics of the interview situation (Abrams, 2010; Thompson, 2000), and in childhood memory research, the narrating adult and the child re-emerging through recollection are always present simultaneously (Fass, 2010). Participants are understood as active co-constructors of their narratives rather than passive sources of information (Abrams, 2010; De Wilde et al., 2020).

The experiences revealed in this study must be contextualized within post-war Finland. The development of Finnish healthcare was delayed by the civil war, the economic depression of the 1930s, and the Second World War (Mattila, 2011). In the early 1960s, the ratio of physicians to population was one per 1,700 inhabitants nationally; in rural areas, this was as low as one per nearly 9,000 residents (Keskimäki, 2022). Polio patients were treated in infectious disease units or central hospitals, with rehabilitative and surgical care available exclusively in Helsinki (Muiluvuori, 2010), meaning that geographical location was a significant determinant of the care experience. Nurses practiced within the prevailing behaviorist philosophy of pediatric nursing of the time (Jolley & Shields, 2009).

### Data Collection and Participants

A combination of semi-structured and dialogical interview methods was employed (Abrams, 2010; Shopes, 2011). The semi-structured interview topics included background information about the participants and their families, their childhood and family life before falling ill, the onset of their illness, the hospital care during the acute phase, their corrective surgeries, the rehabilitation phase, their return home, the impact of illness on their family, and the support of their family. The dialogical interview method invited the participants to bring meaningful objects related to past events, including photographs, diaries, newspapers, magazines, and other memorabilia. During each interview, the researcher and the participant reviewed the objects together, facilitating the recall of past experiences and the activation of sensory memory (Denzin, 2001; Kvale, 2006; Tanggaard, 2009).

The interviews were conducted in Finnish by the researcher (MEK) between September 29, 2018 and June 30, 2019. Forty participants were interviewed, while one participant wrote about their experiences to researcher. The participants (N = 41) were Finnish polio survivors who had undergone corrective surgery and/or demanding inpatient rehabilitation during childhood. The participants included 13 men and 28 women born between 1940 and 1963, with a mean age of 76.4 years at the time of the interviews. The majority were born during the late 1940s and early 1950s, reflecting the peak years of the polio epidemic in Finland. Of the participants, 33 had undergone surgical treatments, while the remaining eight had experienced demanding inpatient rehabilitation periods without surgery. The interviews, conducted in participants’ homes, were audio-recorded and transcribed verbatim. The duration ranged from 35 to 278 minutes, with a mean duration of 89.2 minutes.

The Finnish Polio Association assisted with participant recruitment. The researcher contacted prospective participants by telephone, explained the research’s nature and themes, and provided both oral and written information. The participants were permitted to have a relative or next of kin present during the interview. All the interviews concluded with a discussion of the participants’ present lives and positive life events. The researcher monitored each participant’s emotional wellbeing throughout the interview and, if necessary, was prepared to refer them to a healthcare provider. No participant experienced distress during the interview or wished to withdraw. Each participant was given the opportunity to freely share their experiences, as an expression of mutual trust and empathy is central to oral history research (Strandén, 2009).

### Data Analysis

The data were analyzed using reflexive thematic analysis (Braun & Clarke, 2006; Braun et al., 2022). The analysis followed the six phases described by Braun and Clarke (2006): familiarization with the data; coding and development of preliminary themes; reviewing and further refining the themes; defining and clarifying the themes; naming the themes; and writing the research report. The analysis began with the verbatim transcription of the interview recordings, after which the data were read repeatedly to develop a comprehensive understanding of the material. In the initial phase, the analysis focused on identifying semantic, surface-level content. As familiarity with the data deepened through repeated reading, the analysis progressed toward the identification of latent codes and meanings beyond the explicit content. This transition into the reflexive phase enabled the researcher to place their own role and experiences in active dialogue with the research material, and themes were understood as interpretive accounts shaped by the researcher’s subjectivity and by a situated reading of the data (Braun et al., 2022). Through the reflexive interpretation of both the semantic and latent codes in dialogue with the data, the overarching theme, the main themes and subthemes were constructed (Braun & Clarke, 2006; Braun et al., 2022). All phases of the analysis were conducted by the researcher (MEK).

### Ethical Considerations

A commitment to informed consent, intellectual honesty, and confidentiality was maintained throughout all stages of the research process (De Wilde et al., 2020; Lusk, 1997). The study followed the guidelines issued by the Finnish National Board on Research Integrity (2023). A preliminary ethical evaluation was requested from the Tampere Area Ethics Committee for Humanities, maintained by Tampere University, in 2018 (Request for opinion 18/2018; opinion 31/2018), and the committee’s opinion was favorable. The relevant research permits were obtained prior to data collection. All the participants received both written and verbal information about the research, and written informed consent was obtained before the interviews. All the participants were assigned codes; no names appear in the recordings or transcripts, and the data were stored in a secure, data-protected environment. The participants’ childhoods, illnesses, and treatment histories were explored. Prior knowledge of the population indicated that discussing these experiences carried the risk of provoking vulnerability and psychological distress (Halbmayr, 2009). The researcher continuously monitored participants’ emotional states, offering breaks and the option to bypass questions. No participant became unwell or wished to interrupt the interview.

## Results

**Figure 1.**
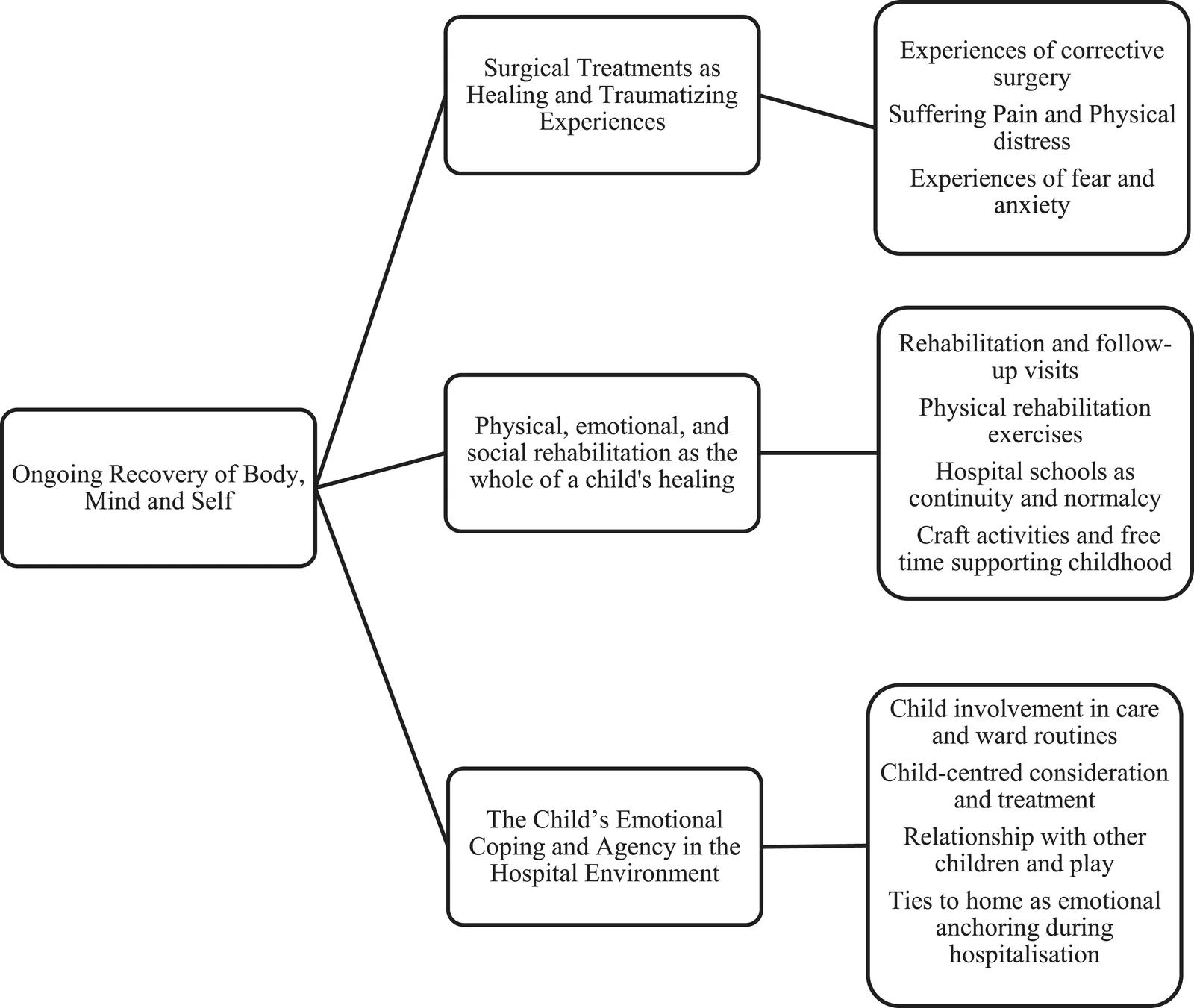
The overarching theme, the main themes, and the subthemes resulting from the thematic analysis

From the thematic analysis, an overarching theme was developed, one that integrated the surgery and rehabilitation experiences of polio survivors: ‘The Ongoing Process of Recovery: Body, Mind, and Self’ reflects children’s recovery as a holistic, continuous process describing the physical, psychological, and social dimensions of those who lived through it, as well as the structures of recovery within the hospital environment and its hierarchy. The participants’ experiences reflected a tension between the healing and fracturing influences of the illness and the hospital environment. This tension is visible as a cross-cutting thread through the three main themes: ‘Surgical Treatments as Healing and Traumatizing Experiences’, ‘The Multidimensional Nature of Rehabilitation’, and ‘The Child’s Emotional Coping and Agency in the Hospital Environment.’ The results are presented under these three main themes. Figure 1 illustrates the experiential world of children constructed using the thematic analysis.

### Surgical Treatments as Healing and Traumatizing Experiences

The theme thus reflects the contradictory nature of corrective surgery—its perceived necessity on the one hand, and, on the other, the experiences of pain, fear, and loss of control over one’s own body linked to it. This theme is reflected in three sub-themes: experiences of corrective surgery, suffering associated with pain and physical distress, and feelings of fear and anxiety. Each subtheme is described in the following sections.

The analysis revealed how the experiences of corrective surgery in children diagnosed with polio constituted a dual phenomenon: The same procedures could offer hope for the recovery of the children’s functional capacity and the normalization of their bodies while simultaneously causing suffering. The theme thus reflects the contradictory nature of corrective surgery—its perceived necessity on the one hand, and, on the other, the experiences of pain, fear, and loss of control over one’s own body linked to it.

The participants’ experienced surgery as simultaneously restorative and traumatizing. Corrective surgical interventions occupied a central and often prolonged role in children’s narratives. The participants described a wide range of procedures, including ankle correction surgeries, tendon transfers, epiphysiodesis (surgical growth arrest), spinal fusion, and knee and hip surgeries. The experiences of corrective surgical treatment did not appear as isolated events; rather, they constituted identity-forming experiences during childhood: on the one hand, physical healing was seen as their desired goal; on the other, the experiences were accompanied by distress and disappointment—as the analysis revealed, surgery was experienced either as restorative and function-enhancing or as unhelpful and experimental in nature. In restorative narratives, children collectively recalled, for example, improved walking ability following surgery or liberation from assistive devices. Surgery’s positive impact was associated with a sense of the body normalizing or recovering:

In the mornings, I remember that at first you were not really allowed to get up from bed at all. It must have been about a week and a half since we just stayed in bed, so it was just rest, there was not much else going on. Then they started trying to get me up, or rather I did manage to get up, but there was no cast on this one, just bandages, and there were proper stitches too. They kept me there until the stitches could be taken out, and then they checked whether the boy could move with it. But it was strange. I was able to walk, and when I came home, I did not need any aid at all, no cane or anything. It healed so well. (Polio survivor 1)

By contrast, some of the narratives reflected the cumulative burden of repeated and prolonged surgical treatments. The narratives were partly constructed around a persistent sense of being perpetually in plaster, preparing for surgery, or recovering from it, with each hospital stay lasting months. Some of them underwent so many surgeries that, even in adulthood, they were unable to recall all the procedures performed—the legacy was a body marked by scars. The narratives conveyed an image of confinement to bed and profound loneliness. Some perceived their own treatment and situation as unfair, particularly when they witnessed other children playing freely. Some associated corrective treatments with intense bitterness and anger, describing that they felt like ‘guinea pigs.’ Such experiences eroded their trust in care and reinforced the sense that bodily interventions were uncontrollable and externally imposed.

They did tendon transfers and all sorts of things. They took a tendon from somewhere and transferred it to the thumb for instance. They carried out these experimental procedures. And then once, one of the doctors grabbed me out into the corridor and said, well we could do something about yours too—nothing is working properly there either, so we will fix that as well. But you always had to go there, there were senior consultants and other doctors, and they would all look together and decide whether to operate on something. (Polio survivor 10)

The participants’ experiences were also marked by intense distress related to post-operative nausea and vomiting. The pain experiences varied, and some narratives reflected a sense that analgesic medication was given sparingly, even when pain was intense. Pain appeared to be undertreated and left for children to bear alone. Morphine was frequently mentioned as the pain relief provided following knee surgery and other procedures; however, its significance was ambivalent. It brought relief from pain but simultaneously produced confusing and unpleasant side effects. Some described the sensation the medication produced as strange, saying they felt ‘as if in a cloud.'

The participants recalled that physical discomfort and pain were also caused by various assistive devices, supportive apparatuses, and stretching procedures. The Milwaukee brace caused skin breakdown, adding both pain and discomfort during the recovery phase. The post-operative stretching of operated limbs proved to be a particularly distressing experience. This procedure was performed without pain relief, and in some memories, the absence of medication was again a prominent feature. According to one participant, the stretching treatment caused a serious complication when the wound of the limb being stretched opened.

I remember the doctor stretching the limb. No pain relief was given at all, and it was terrible. After the surgery, when the stitches were taken out, the doctor would stretch it and I screamed terribly. I remember it so well, even my fellow patient said afterwards that she had thought, how awful. And then suddenly there was a tear in this left one, and I said that the wound had opened. The stitches were already out. The doctor said it could not be, but it was, the stitches had come apart, and the wound was weeping and festering. (Polio survivor 34)

Corrective surgical treatments were also associated with a broad range of other fears and anxious experiences. For some, fear extended beyond individual procedures to encompass the future and the unpredictability of the treatment trajectory, while some feared, on each visit to the hospital or outpatient clinic, what new procedure would be devised for them and what surgery they would have to undergo again. Hospital equipment and the clinical environment could also appear threatening. X-ray machines were described as frightening, and anesthesia experiences were recalled as particularly traumatic. Ether anesthesia was repeatedly recalled as deeply distressing. During ether anesthesia, some participants described a sensation of being unable to breathe or of dying, as the procedures had not been explained to them in advance. One participant described how they were asked to choose the method of anesthesia themselves (injection or mask), adding a burden of responsibility to an already deeply frightening moment.

The anesthetics were traumatic experiences. Back then, they would ask you whether you wanted an aesthetic injection or a mask an aesthetic, and yes, they did ask the child. Well, of course a child does not want an injection, so I remember choosing the mask—and it was just awful when they put the mask on and you felt like you could not get any air. You breathed as hard as you could, but no air came. There was this terrible panic just before you went under—you fell asleep quickly enough, but it was a horrible feeling, being held down with the mask forced on you and not being able to get any air. (Polio survivor 11)

Frightening details of various procedures were also described precisely. For example, cast removal with an electric saw was described as frightening because no one had explained what the saw would be used for. Many participants also described how treatment experiences manifested in their dreams. One participant described recurring dreams in which water pulled them under. Moreover, the analysis built a picture of how the serious conditions or deaths of other children were experienced as frightening. Fear of what might happen to oneself also manifested as a child’s fear of a priest or of being on a hospital balcony. The precise recollection of such memories in adulthood may be indicative of trauma formation during the hospitalization period.

And then I always had this same recurring dream, every night, a hand would rise from the toilet bowl, a black hand, and I could not escape it, it just kept coming after me. And then I would jump into, there was an indoor pool at the hospital, I think there was only a little water in it, knee-deep or so, and I would jump into that pool, but the pool just pulled me deeper and deeper and the hand followed me down, until I reached a certain floor and there was an operating theatre, and I had to go into that operating theatre because otherwise the hand would catch me—the operating theatre was the salvation, I had to go in there. The same dream kept coming back every night. It has stayed with me. (Polio survivor 11)

For some participants, the post-operative recovery phase included distressing experiences regarding eating. After surgery, they had no appetite, yet nurses sometimes force-fed them and threatened tube feeding. Force-feeding was described as distressing and controlling, undermining the participants’ sense of autonomy and reinforcing feelings of insecurity.

### Physical, Emotional, and Social Rehabilitation as the Whole of a Child’s Healing

This theme is organized by the central concept that rehabilitation was experienced not merely as a series of clinical interventions but as a prolonged, institution-bound process shaping every dimension of children’s daily lives. Five subthemes characterize this theme: rehabilitation and follow-up visits, physical rehabilitation exercises, hospital schools as continuity and normalcy, craft activities and free time that support childhood, and experiences of corrective surgery. Each is discussed in the sections that follow.

The analysis revealed how rehabilitation was reflected in the participants’ experiences as a multidimensional phenomenon, in which rehabilitation was not a singular or uniform process but one in which the physical, educational, and social dimensions of childhood were interwoven. The analysis further constructed an understanding that for the participants, rehabilitation represented both a medical necessity and a lived reality that shaped their sense of self, their relationships, and their understanding of their own bodies, as well as their capacity to preserve the conditions of an ordinary childhood within a prolonged hospital environment. By contrast, rehabilitation also brought children security during their hospital stay, a sense of normality, and enabled play and participation, which, in turn, strengthened the experience of being a child.

Follow-up checks and assessments were often conducted in the capital region and in specialist units for people with physical disabilities. Follow-up appointments could continue for years, at six monthly intervals or over a period of up to ten years after procedures. These visits also held social significance, giving rise to friendships. There were also experiences of children travelling alone by train to the follow-up appointments, although a relative would be waiting upon arrival. In addition to monitoring physical recovery, one participant also recalled psychological testing as part of the follow-up visits.

The follow-up appointments were the only visits, and even there they just checked things—as you can see from the records, they took an EEG and a chest X-ray and skull and thorax X-rays, and measured blood pressure, checked the eyes, tested the IQ, did the Rorschach and all of that. I remember the inkblot tests too, those were done. Apparently, they were checking that there was nothing wrong up here. (Polio survivor 12)

At the same time, the follow-up visits were psychologically burdensome for many. During each visit, they feared that they would be made to stay back in the hospital. The visits also produced strongly embodied experiences of shame and vulnerability, as some children were asked to walk before doctors and nurses, sometimes partially undressed, and to be photographed. Particularly traumatic was the experience of seeing oneself in a mirror while multiple professionals assessed one’s walking performance. Witnessing another unclothed child walking could also evoke shame and embarrassment. Moreover, in the waiting areas for appointments, children compared themselves with others who had even greater mobility impairments, provoking confusion and distress. Anxiety was heightened when adults discussed the child’s illness and prognosis within earshot without explaining anything to the child, thereby reinforcing children’s uncertainty and insecurity, as well as their imaginings about the course of the illness.

They would always walk you around and move you from place to place. And about this walking when I went there for surgery, they would walk you around in this room so they could see how you stepped and how you walked. Without clothes, and there were a great many people there, many doctors and many nurses. And then we were photographed. (Polio survivor 29)

Physical rehabilitation constituted a broad and multifaceted domain in participants’ experiences, encompassing both active therapeutic practice and children’s own assumption of responsibility for their recovery. The nature and extent of rehabilitation varied considerably. Some described the absence of active rehabilitation, experiencing recovery as occurring through their own natural liveliness and spontaneous movements. Based on the interpretation of the analysis, in such cases, children’s innate curiosity and activity appeared as compensatory factors for the lack of formal rehabilitation. By contrast, for some participants rehabilitation was characterized almost entirely by prolonged bedrest, with physical rehabilitation reduced to months of confinement to bed; being confined to a room was described as profoundly boring, with children’s natural need for movement going unsupported, as they were not even taken anywhere in their beds. The analysis produced an understanding that rehabilitation and assistive devices could simultaneously evoke both positive and negative feelings in children, with exercise sessions being enjoyable on the one hand and demanding on the other, bringing joy or provoking fear.

Yes, back then as I said, we really did rehabilitate. To me it felt like almost a full-time job—there were all sorts of things going on throughout the day. There was physiotherapy on the bench, on the couch, and then there were walking exercises and water exercises, and there was a lot of activity going on. You just always worked on fitness and strength, which was what you trained, and you always had to do as much as you could manage. It really was like proper work. (Polio survivor 10)

The rehabilitation exercises were diverse, addressing the strengthening of overall bodily function through gymnastics, wall bars, walking practice, and relearning to walk through crawling. The physiotherapy experiences ranged from positive to burdensome: some described the physiotherapist as encouraging and motivating, while others experienced rehabilitation as hard work that at times felt commanding and unpleasant. Hydrotherapy and pool-based treatment were frequently part of rehabilitation and were experienced by some as enjoyable and by others as frightening.

I have this image in my mind of a huge steel swimming pool, and in that pool we were given some kind of treatment or did exercises. To me it was great fun, because I had never been anywhere other than a lake before, but now there was warm water and you could splash around in it, and you felt that there was still some strength in that leg after all. (Polio survivor 12)

Rehabilitation stays generally lasted around a month, and in some cases, the episodes were scheduled during the summer to minimize disruption to schooling. When mobility began to return, children experienced this as a cause for celebration. Assistive devices, including metal leg supports, crutches, wheelchairs, splints, and corsets, featured prominently in the data. For some, these gave their weakened bodies a sense of security; for others, they were a source of fear.

And then when it was taken off me, it was such a wretched feeling. It felt like my body was upright, am I just going to collapse completely. You had got so used to it holding your posture properly like this. And then when it was no longer there around you, it felt like I was just falling apart without it, because I did not have it anymore. (Polio survivor 2)

Some experienced rehabilitation as a lifelong institutional commitment. While some spent extended periods in a hospital—for example, 18 continuous months—others were in rehabilitation facilities for three years and returned home only for holidays. Physical rehabilitation thus encompassed, alongside experiences of recovery, also experiences of separation, loneliness, and a childhood structured around the rhythms of the treatment institution.

And then I had a great many operations. Probably something like ten or more before I turned 15. All kinds of tendon transfers, sympathectomies to try to improve the circulation in the worse leg, and—I do not even know. All kinds of bone operations, the ankle was fused at some point because my ankle bent into hyperextension. But there was always a cast on my leg, or I was just recovering from an operation or about to go into one. It felt like that was my childhood. (Polio survivor 24)

Library access and hospital schooling formed an integral part of the participants' everyday life. The hospital school was a central element of their routine during rehabilitation and hospital stays, supporting educational continuity throughout prolonged treatment periods. Attendance at the hospital school was maintained, and learning was facilitated even when functional capacity was limited. The analysis indicates that the hospital school thus represented an effort to maintain children's learning and development in a context where all other aspects of life were dictated by treatment procedures. In this way, the hospital school provided children with a sense of security and normality. The children's hospital, for instance, was described by the participants in particularly positive terms. One participant remembered it as a paradise, filled with wonderful staff, and as a natural part of their daily life.

Well, for a country girl that was really just like being in heaven, it would have been paradise, and of course the food was different than at home, it was regular, there was breakfast and lunch and a snack and dinner and an evening snack, and then there was fruit too. (Polio survivor 12)

The analysis considered craft activities and free time as a central dimension of children’s psychological and social wellbeing. The participants highlighted daily activities, with craftwork, drawing, reading, and playing games as common pastimes. Some described craftwork as more enjoyable than physiotherapy exercises. The role of the play worker (‘play aunt’) was particularly significant: the play worker engaged and activated children, brought joy to their daily life, helped them with homework, and provided them with treats, especially for those who had no visitors.

There was this trolley that came around with all sorts of craft things on it—some modelling clay, very simple things, some books. A trolley like that went around the rooms. She was called the play aunt. (Polio survivor 25)

Craft activities and free time were thus not merely ways of passing time but pathways to participation, meaning, and emotional support within the treatment environment. Excursions to an amusement park, a theatre, and a confectionery factory were recalled fondly, and small everyday pleasures, such as watching television, building a model railway set, or receiving a banana for the first time in their life, underscored the exceptional nature of the hospital environment and the intensity of specific sensory memories.

And then for pleasant memories, we went with the nurses to the chocolate factory once. You got to take sweets home, these little sweet houses to take with you. And you could eat as many sweets as you liked there at the factory. And then there was a trip to an amusement park with the nurses. And the theatre has stayed in my memory too; we went to the theatre as well. I remember some performance where there were witches, and I wondered at how they were depicted or shown, how they flew across the stage, those witches. Those were nice memories. (Polio survivor 11)

### The Child’s Emotional Coping and Agency in the Hospital Environment

This theme was created around the understanding that hospitalized children were active agents who developed ways of navigating, enduring, and, at times, resisting the institutional environment. The analysis generated a picture of how children’s emotional coping and agency were also shaped by the degree to which they were acknowledged and included in their own care. Further, the analysis constructed the understanding that the hospital environment was simultaneously a place of vulnerability and resilience for the participants, where the conditions for coping were found not only in professional care but in the everyday structure of their ward life, peer relationships, and the nurturing of ties to home. Four subthemes characterize this theme: child involvement in care and ward routines, child-centred care and treatment, relationships with other children and play, and ties to home as a source of emotional anchoring during hospitalization. Each is discussed in the sections that follow.

The participants’ narratives revealed child-centered care as a contradictory experience: on the one hand, children experienced moments of safety, attachment, and genuine care; on the other, they encountered institutional routines, hierarchical structures, and the dismissal of their emotional experiences. The hierarchy of the hospital environment was present in every narrative. Ward rounds, for example, were experienced as authoritative events, and nurses were described as fearful of doctors. The analysis thus constructed a picture where children’s daily lives were tied to hospital routines and rules, which created predictability and a sense of safety for some and a sense of their autonomy being constrained for others.

They were these authority figures who came round, perhaps once a week or so. Everything had to be spotlessly clean, the beds had to be perfectly made without a single crease in the covers, and not a speck of dust anywhere in the rooms. They came in like kings and queens, and went around from bed to bed, talking over you. (Polio survivor 30)

The participants memories of nursing staff were both positive and negative. In many narratives, nurses were described as kind and reassuring, doing their best to help and attend to children’s needs. As the analysis progressed, the experiences of ‘motherly’ nurses became particularly significant; these nurses were described as ‘named nurses.’ These named nurses, as per the analysis, were acting in a sense as surrogate mothers, offering children something extra in the way of care, such as bringing honey water to a child with a cough, cleaning under a child’s fingernails, or letting a child sit in their lap. In the participants’ minds, these extra gestures registered as concrete signs of safety and being cared for. Doctors, too, appeared as father figures as well as authority figures, with the participants using words such as ‘fatherly figure’ in their narratives. Moreover, the adult patients who showed children affection through treats and small gestures also extended the care children received and gave them a sense of being seen.

I remember those old doctors who were so kind and fatherly somehow. They were all men, those orthopedic surgeons, and I remember them because the prosthetics workshop was in the same building at the time. It was down in the basement of the same building, and those men, well they seemed like uncles to me back then. They wore brown coats, and they were wonderfully warm, like father figures and all that. As a child you probably seek that kind of security from anyone who has that sort of fatherly quality. And then there was a nurse there too, it seems I must have had some nurse when I was very small who was a kind of mother substitute for me. (Polio survivor 31)

At the same time, some participants also described instances concerning lack of empathy, physical punishment, and shaming children. Moreover, the children's complaints were not always acknowledged, with the nursing staff advising children that a 'good child' was one who did not complain. Some participants highlighted that some nurses slapped children on their face or buttocks; some were even scolded harshly when they were unable to move and soiled themselves. While children felt lonely without nurses caring for them, some nurses interpreted a child's crying as a sign of being 'spoiled' or 'difficult.' Children's emotional expressions were thus evaluated through the lens of discipline and obedience rather than from the perspective of their emotional needs.

But then I remember crawling around on the floors of the hospital too, and being… yes, quite resistant as well, I remember that. I had of course been defiant and difficult, and I remember the nurses saying that you will be taken to another hospital, they used to threaten me with something about another hospital… perhaps I somehow understood that they were talking about a psychiatric hospital or something like that… for this reason, a test was carried out which established that there was nothing wrong with the patient's intelligence. I must have been just under five years old. (Polio survivor 17)

Children's ward life was organized around daily routines: They were expected to make their beds and keep their belongings tidy, which served to support their functional capacity and as a disciplinary practice. Some participants described involvement in ward tasks, such as meal distribution, as a source of pride and meaning, while the same routines reinforced a sense of institutional control for others.

And then when I was a little older, I was always allowed to help the ward assistants with the meal rounds. I could take the trays, I was not allowed to carry them to the patients myself, but I could always hand them over. That was an important task. I loved it. (Polio survivor 12)

Children’s participation in understanding and decision-making about their own care was frequently insufficient: procedures were not explained, medical information was withheld, and conversations were held in children’s presence as though they were invisible. Their agency manifested as resistance, including demanding discharge, refusing assistive devices, and concealing food, all of which can be understood as responses to pain, fear, homesickness, and the loss of autonomy in a hierarchical and routine-bound environment.

I was very small and a poor eater, and I always threw all the food I did not want out of the window, and there was probably quite a lot of it. Then someone came and asked whether someone had been sick out here. Or perhaps they realized it was me who had been throwing it. Then because I was such a poor eater, I was given cod liver oil, and I fed it to the other girl in the room who had not been given any, and I told her it was for her. And then somewhere in a nearby room there was an adult patient, and she had chocolate bars on her bedside table. She was asleep, and I stole a chocolate bar from her and got caught. But I was not punished at all. (Polio survivor 16)

It is noteworthy that some children were delegated to supervise their peers and even permitted to restrain poorly behaved children, illustrating both the disciplinary nature of ward culture and the ambiguous dimensions of participation.

And then two of the older children were given authority, if someone would not settle down to sleep, they were allowed to tie them to the bed, because we had these kinds of braces, so they had permission to tie them to the bed if someone was not behaving properly. (Polio survivor 11)

According to the analysis, the relationships between children were found to be a central coping resource, although they also encompassed bullying. Extended hospital stays gave rise to children’s own communities and peer groupings, within which children learned both how to be good and how to be unkind to each another. The participants described a sense of community expressed through playing games together, chatting during evening gatherings, and entertaining each other. Racing wheelchairs in the corridors with friends was described as joyful, and according to some accounts, lifelong friendships were formed in the hospital. Follow-up appointments could also be occasions for making friends, and seeing peers reinforced a sense of solidarity and shared identity.

Play and imagination, it can be suggested from the analysis, functioned as mechanisms for regulating the feeling of safety and alleviating the monotony of hospital life. Children told each other stories, sang together and played pranks, threw paper airplanes at roommates, and pushed others in their beds or wheelchairs and shared jokes. The shared games represented mutual survival, and shared resistance against ward routines produced a momentary sense of freedom.

Me and another child were out on the balcony, and then suddenly the idea came to us, let's pull a trick. We rushed inside at full speed and told everyone that Sputnik was passing overhead. Everyone came rushing out to the balcony. Of course there was nothing to be seen, but we said it had just gone past. They never believed us, and it was never set straight. (Polio survivor 10)

Imagination occupied a significant role in the participants’ narratives, taking the form of storytelling, shared daydreaming about escaping, and pranks against ward routines that produced a momentary sense of freedom. The narratives also conveyed images of empathy and a home-like emotional atmosphere among children: The older children served as parental figures for the younger ones, and the younger children sometimes sought consolation and physical contact from their peers than from the nursing staff.

And then these strange experiences that stayed with me from childhood—every evening, always at the same time, someone would tickle the soles of my feet. It went on for a long time, I always knew that when the lights went out and it had been dark for a little while, someone would sort of tickle my feet. And then one time I turned around so that my legs went the other way, with my knees towards the head of the bed, and then whatever it was tickled my knees. It was somehow such a strange sensation, I do not know what it was—whether it was psychological or what. (Polio survivor 11)

At least one boy I remember, though I do not know his name or anything else about him—we would all go out into the corridor and sit side by side, and there was the evening devotion. And there was always this one little boy who wanted to come and sit in my lap. (Polio survivor 39)

The bond with home seems to have constituted a central dimension of children's emotional coping and simultaneously represented a fragile bond that the hospital institution could either strengthen or weaken. Experiences of intense homesickness were present. At the same time, prolonged absence could cause the idea of home itself to fade, manifesting, for example, in a child failing to recognize their mother immediately upon her arrival.

I remember one time when my mother came. I do not know which hospital stay it was. I was not entirely sure whether it was her who this person was. But in some way, I sensed that it was my mother. And I remember that the memory of home faded, or it became unclear, there was no longer a clear picture, and it felt like it had been a dream, had I been somewhere, the memory of home grew hazy there in the hospital. (Polio survivor 11)

Visits and contact with home were often limited. The visiting hours could end up in disappointment if there were no visitors. Sometimes parents were unable to visit at all during a hospital stay or came only once every couple of months; contact with home was sometimes maintained through visits from relatives. In one narrative, parents were actively encouraged to ‘forget’ the child, an instruction that contributed to a recurring experience of abandonment, particularly as the child moved between multiple hospitals and repeatedly lost contact with their parents and friends.

Letters, telephone calls, and parcels represented a tangible connection to home. However, the hospital’s control could undermine this bond: Nurses rationed treats, and the gifts sent to individual children were sometimes distributed among all the children on the ward. Personal objects, including toys and clothing, served as anchors of children’s identity and their relationship with home, and their loss or redistribution was experienced as a significant violation.

We had this big hall where everyone had cot beds, and there were quite a few of them—I would not dare say exactly how many, but it was a big hall, and all the children had different kinds of disabilities. Then when they cried at night, as I said, they (nurses) would come and fetch my teddy bears and toy cars to calm them down. And as soon as they fell asleep or the nurse left, I would go and take them back. (Polio survivor 42)

## Discussion

The overarching theme ‘The Ongoing Process of Recovery: Body, Mind, and Self’ captures children’s recovery as a holistic and deeply lived process. Corrective surgery and rehabilitation were not experienced as isolated clinical events but as a prolonged, multidimensional process that gradually reshaped children’s sense of self. Recovery extended far beyond physical healing, encompassing body image, psychological wellbeing, and social experience. This interpretation aligns with nursing science literature, which describes identity change in chronic illness as a process of becoming whole again through meaning-making and social relationships (Hajdarevic et al., 2024), and with qualitative research on polio survivors, which demonstrates that early experiences of immobilization, painful treatment, and separation from home necessitated the development of psychological coping strategies that could become maladaptive in later life at the onset of post-polio syndrome (Sjödahl Hammarlund et al., 2021).

The central finding is that surgical treatments carried dual significance: while they were necessary and function-restoring, they were also highly distressing and potentially traumatizing. Pain, physical distress, procedural fear, and anxiety shaped how children remember surgery and how it became embedded in their broader experience of recovery. This is consistent with international research, according to which invasive treatment events may trigger posttraumatic stress-like symptoms in children and families (Kazak et al., 2015), perioperative anxiety is associated with the intensity of postoperative pain (Chieng et al., 2014), and surgical procedures may leave significant psychological sequelae for both children and parents (Stanzel & Sierau, 2021; Turgoose et al., 2021). These experiences must be understood in their historical contexts. The Finnish children’s experiences of repeated surgery, inadequate pain relief, and bodily subordination reflect the structural conditions of an era in which children’s subjective experience remained peripheral to treatment decision-making.

The findings indicate that children suffered both emotional and physical harm during treatment—harm that was at times intentional and at times inadvertent. In this study, participants’ experiences of intentional harm were tied to their identities as children living with disabilities, resulting in harsh discipline and force-feeding. Their experiences of inadvertent harm included the pain and fear caused by treatments they perceived as experimental. Research has shown that the treatment of polio patients was callous in the 1940s and 1950s, with no emotional support provided and ties to home left unnurtured (Schanke et al., 1999). The children adapted to appear unrelentingly upbeat, learning to please others and to ensure that nursing staff and families could celebrate their rehabilitation, a performance that concealed profound suffering (Schanke et al., 1999). This illusion of cheerfulness was reinforced by the prevailing view that polio survivors were expected to walk again, even when this was not always realistic, arising from a disability culture in which disability was seen as a source of shame and people living with disabilities were expected to be made fit for society as quickly as possible (Wilson, 2005).

In the present research, children’s narratives in which the experience of being a ‘guinea pig,’ force-feeding, and bitterness at unfair treatment appeared as accounts in which medical practices silenced their lived experiences and subjugated their bodies to external authority. These experiences can be understood through the Foucauldian framework of bodily objectification outlined by Yoshida and Shanouda (2015), in which the disabled body is subjected to medical and rehabilitative discourses that silence children’s lived experiences. In the present research, this silencing operated on multiple levels: the treatment procedures were not explained, children’s emotional expressions were evaluated through the lens of discipline rather than care, and children’s own accounts of pain and fear were systematically dismissed. Children’s resistance, such as throwing food out of windows, demanding discharge, and retrieving confiscated toys, can be read not merely as behavioral non-compliance but as embodied assertions of selfhood against a system that treated the body as an object of intervention rather than as a subject of experience.

In this study, rehabilitation was found to be a prolonged process, with some children spending up to three years in rehabilitation facilities. During extended hospital stays, free time, play, and imagination played a significant role in building a sense of security. This finding is supported by international literature demonstrating that play and activity-based interventions reduce children’s stress and support recovery during hospitalization (Gjærde et al., 2021) and that in-hospital education supports developmental continuity and facilitates reintegration into mainstream schooling (Burns et al., 2021). The significance of hospital schooling identified in this study connects to a broader historical pattern documented in research on polio and education. Polio profoundly disrupted children’s access to schooling, with many children excluded from mainstream education during and after treatment (Altenbaugh, 2006). In the present study, hospital school represented a counterforce to this disruption: It provided educational continuity, a sense of normality, and a structured daily rhythm within an institution otherwise organized entirely around medical procedures. That participants recalled the hospital school in predominantly positive terms—and in some cases described the children’s hospital as a paradise—suggests that educational provision functioned not merely as academic instruction but as a form of psychosocial protection within a depriving environment.

The findings also reflect the rehabilitation philosophy operating in Finnish pediatric care during the 1950´s-1960´s. While the Kenny method based on active patient participation had begun to transform rehabilitation practice internationally in the early 1940s (Neumann, 2004; Rogers, 2021), it was not adopted in Finland; the nursing textbooks of the 1950s and 1960s did not recommend it, and treatment remained nurse-centered and symptom-based (Veltheim et al., 2024). This absence of a patient-active rehabilitation philosophy may partly account for the variation in rehabilitation experiences described by the participants, ranging from prolonged enforced bedrest to intensive physiotherapy, and for the limited agency children had over their own recovery. In this research, the meaning of assistive devices proved to be contextual. Previous research has shown that orthoses could be experienced primarily as symbols of disability and sources of social stigma (Sjödahl Hammarlund et al., 2021), whereas in the present study, corsets and metal splints could also be experienced as providing a sense of security for a weakened body.

The findings also showed that children’s agency was expressed not only through cooperation with treatment but also through adaptation, resistance, emotional regulation, and coping mediated by peers and connections to home. Power structures, ward routines, restricted visiting hours, and limited family contact profoundly shaped children’s sense of safety and control. Viewed through family-centered care frameworks—in which dignity and respect, information sharing, participation, and collaboration are core principles—the situations described in this study—where children’s participation was restricted and information withheld—constitute significant risk factors for psychological safety. Previous research confirms that family-centered care models are associated with reduced parental anxiety and improved communication, although no model has yet provided continuity across all phases of care (Curtis et al., 2016).

The experiences of the polio survivors strongly reflected children’s insignificance in the eyes of adults. The questions concerning children’s needs and hopes were not systematic. The Finnish hospital system was being modernized in the 1950s and 1960s, and children’s medical care was only beginning to develop after the Second World War. Children were initially treated alongside adults and did not have any special status in hospital care, which was vividly reflected in the narratives of the research participants. There was also a shortage of nursing skills in Finland at the time (Muiluvuori, 2010), and the nursing culture globally had only just begun to develop competence in family-centered care (Jolley & Shields, 2009). The research findings support previous research that has indicated that children living with disabilities have very little control over their bodies and treatment (Yoshida & Shanouda, 2015).

### Strengths and Limitations

This study has several limitations that should be considered when evaluating its findings. Although interviews provide valuable research material, people tend to recount what they consider worthy of sharing. The purpose of the study was not to produce a generalized oral history but to highlight children’s experiences of illness and treatment, which can complement the broader history of nursing and medicine.

The sample is homogeneous in terms of its historical context: All the participants were Finnish polio survivors who contracted the disease during the epidemic years of the 1940s and 1950s. This is a contextual feature of the study that simultaneously limits the transferability of its findings to other national or cultural settings. Sex-differentiated analysis was not among the study objectives; nor was the sample large enough to support a systematic comparison between sexes. Women were considerably more represented than men in the sample (28 women, 13 men)—which may reflect self-selection or sex differences in the willingness to participate in oral history research—and this should be taken into account when interpreting the findings.

From the perspective of oral history research, the method has both strengths and limitations. As a strength, oral history captures experiences that are not recorded in official documents: Children’s subjective experience of hospital care is precisely the kind of tacit knowledge that remains in the shadow of the official history of medicine and nursing. The dialogical interview method, with its use of memorabilia, activates sensory memory and produces richer data than interviewing alone, and the material complements the official history of medicine and nursing from the patient’s perspective. As a limitation, it must be noted that oral testimony is constructive in nature: Memories are shaped by later experiences, accounts heard from others, and collective social memory, and the boundary between individual memories and collective memory cannot always be clearly drawn. Furthermore, self-selection may influence the data: Those who wish to share their experiences may differ from those who do not, particularly with regard to traumatic experiences. The interview situation itself also shapes what is recounted and how.

The participants narrated events from decades ago, making accurate recollection uncertain. It cannot always be determined whether the narratives were based on the participants' own memories or on accounts learned from others over time. Their memories may also have been affected by the psychological suppression of painful or traumatic childhood events, and translations may not fully capture the linguistic richness of the original Finnish expressions. The participants were invited to bring memorabilia to the interviews, such as photographs, toys, and treatment records, which played an important role in activating memories.

The trustworthiness of the analysis conducted by the researcher was strengthened by returning regularly to the original data throughout the entire analytical process, ensuring that the interpretations remained grounded in the data. A research diary and analytical memos were maintained throughout the research process, supporting reflexivity and the documentation of analytical decisions (Braun et al., 2022 ). The research generated information about the historical treatment experiences of significant social relevance in Finland. This research report was produced in compliance with responsible scientific practice and is not intended as a critique of past events (Boschma et al., 2008). Instead, it aims to examine those experiences in the context of the prevailing culture of care at the time.

## Conclusion

The contribution of this research to the historiography of nursing is significant. It illustrates how the nursing culture of the 1950s and 1960s constructed the position of the child patient hierarchically and institutionally in a context in which obedience, control, and the restriction of information were—to a considerable degree—accepted and even normative practices. Examining these experiences is important because it reveals the historical coexistence of care and discipline: The hospital was not merely a place of healing but also an educational and moral system in which childhood was defined through rules and power. Bringing patient experiences into historical scholarship produces ethical memory and corrects the dominant narrative of medical progress by making visible the cost of care for those who lived it.

Future research must explore how childhood treatment experiences are reflected in later life trajectories, engagement with health services, and identity formation and identify the specific nursing interventions that prevent the harmful psychological sequelae of care. Research on polio survivors’ long-term adaptation has shown that the strategies developed in childhood, such as optimistic thinking and trust in one’s ability to manage, can remain useful in later life. At the same time, the historical experiential perspective offers healthcare a critical mirror: Understanding the practices of the past helps identify the vulnerable structures of contemporary systems. Research of this kind is therefore not merely a description of the past but essential knowledge for building more human, participatory, and holistic care. It is also possible that the treatment experiences in childhood are reflected in later life phases, influencing how individuals relate to healthcare, seek care, and experience trust in healthcare professionals and the healthcare system.

## Data Availability

The data that support the findings of this study are available from the corresponding author upon reasonable request.*
